# Collapsed membranes within pelvic cyst: What is the diagnosis?

**DOI:** 10.1002/ccr3.767

**Published:** 2017-01-17

**Authors:** Seyed Ali Dehghan Manshadi, Omid Rezahosseini, Sadegh Saberi, Faeze Salahshour, Shahrzad Mahdavi Izadi

**Affiliations:** ^1^ Department of Infectious and Tropical diseasesImam Khomeini Hospital ComplexTehran University of Medical SciencesTehranIran; ^2^ Resident of Infectious DiseasesDepartment of Infectious and Tropical diseasesImam Khomeini Hospital ComplexTehran University of Medical SciencesTehranIran; ^3^ Department of Orthopedic surgeryImam Khomeini Hospital ComplexTehran University of Medical SciencesTehranIran; ^4^ Department of RadiologyImam Khomeini Hospital ComplexTehran University of Medical SciencesTehranIran; ^5^ Resident of PathologyDepartment of PathologyImam Khomeini Hospital ComplexTehran University of Medical SciencesTehranIran; ^6^ Universal Scientific Education and Research NetworkTehranIran

**Keywords:** Albendazole, echinococcosis, hydatid cyst, pelvis

## Abstract

Collapsed membranes and daughter cysts are pathognomonic for hydatid cysts on imaging. The comma‐shaped lesions, visible within the hydatid cyst in sagittal view of MRI, are collapsed membranes. Although primary hydatid cyst of pelvic cavity is rare, clinicians should remember to include hydatid cysts in differential diagnosis of pelvic cysts.

A 53‐year‐old woman was referred to our hospital with left buttock pain for 1 year. The pain progressed gradually. The point of maximal pain was on the posterolateral aspect of the left buttock. Her pain radiated to her thigh and increased with walking. No superficial lesion was obvious upon physical examination. No recent history of injections was found, and the patient had no history of fever or weight loss. Pelvic MRI revealed a cystic lesion in the inferior pubic ramus, ischium, and iliac bone, extending to the groin, buttocks, and posterior compartment of the upper thigh. In addition, the lesion had extended to the pelvic cavity via iliac bone cortical defect. Small daughter cysts and collapsed membranes within the mentioned cysts were seen. A tissue biopsy was obtained by CT‐guided biopsy, and diagnosis of the echinococcal cyst was confirmed by histopathology. No visible cysts were found in the liver or lungs.

Hydatid disease is a zoonotic parasitic infection caused by Echinococcus sp. Human beings can be infected as an intermediary carrier upon eating unwashed vegetables when parasite eggs are swallowed. The parasite embryo gets into blood circulation from the human intestine and can enter every organ. It most commonly involves the liver or lungs, and isolated pelvic involvement is rare 1, 2. Collapsed membranes and daughter cysts are pathognomonic for hydatid cysts on imaging. The comma‐shaped lesions that are visible within the hydatid cyst in the sagittal view of the thigh are collapsed membranes. Treatment began with albendazole tablets 400 mg twice daily. Due to the complexity of the lesions, further surgery was not performed (Figs 1, 2, 3, 4).

**Figure 1:**
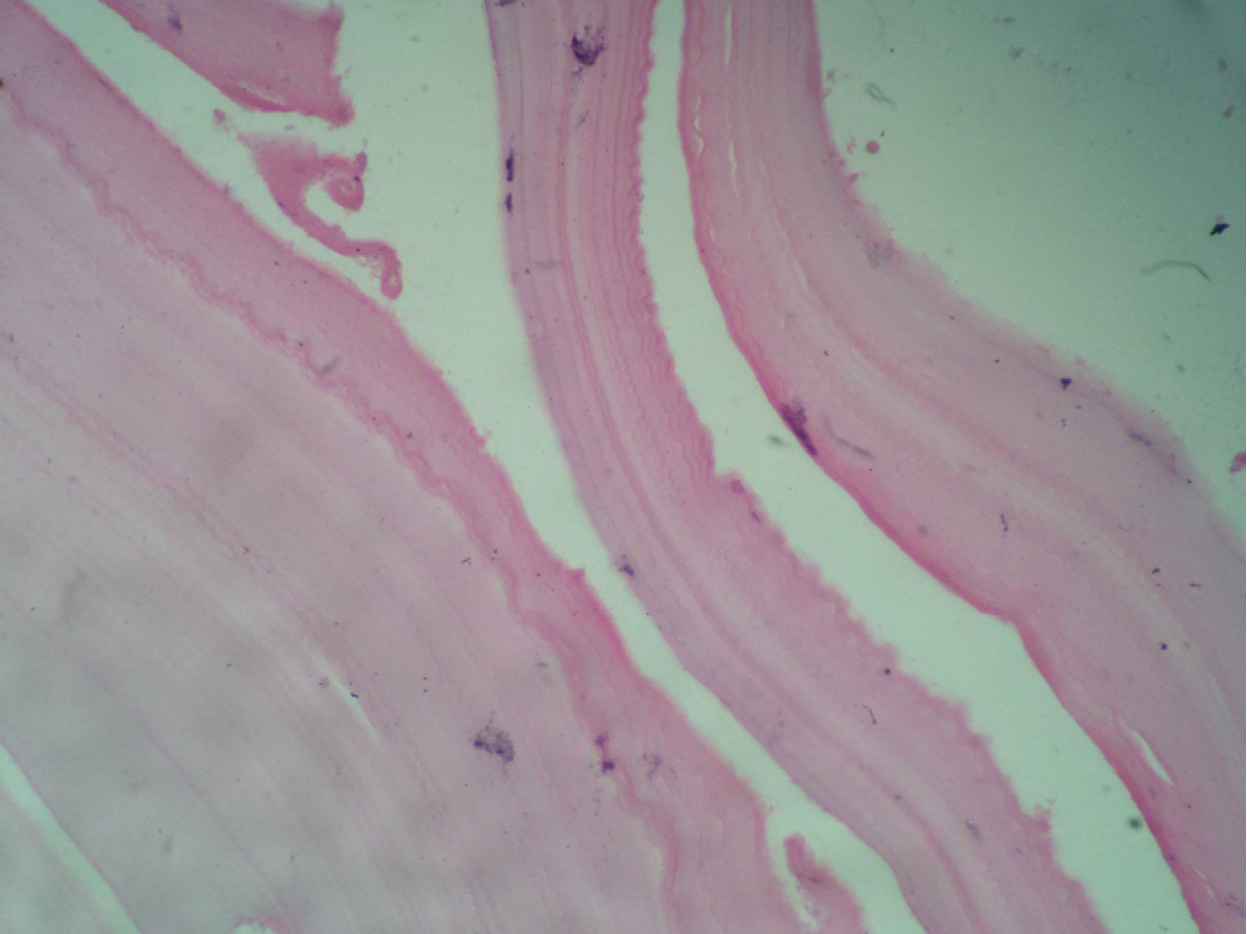
Microscopic view of hydatid cyst layers.

**Figure 2:**
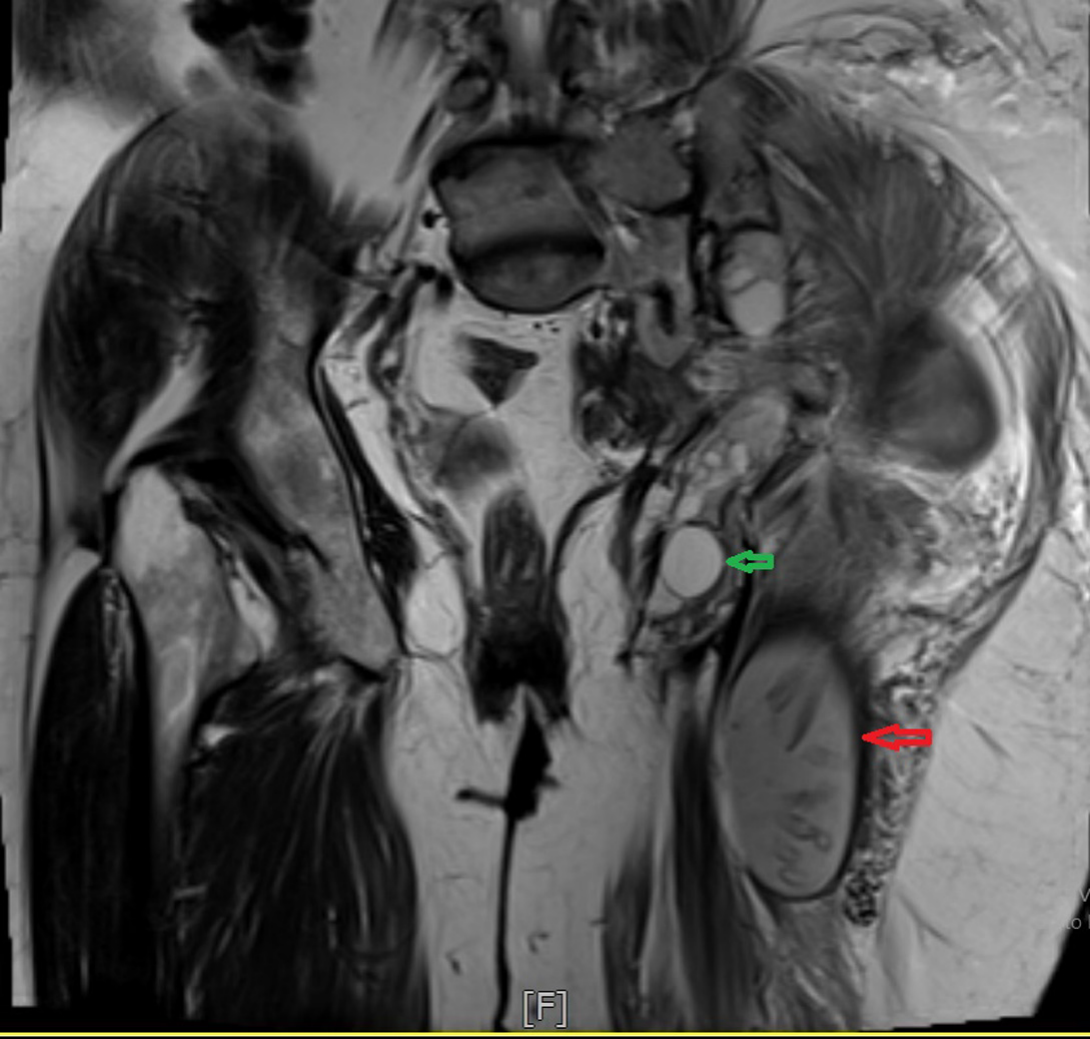
Hydatid cyst (Red arrow) and daughter cysts (Green arrow).

**Figure 3:**
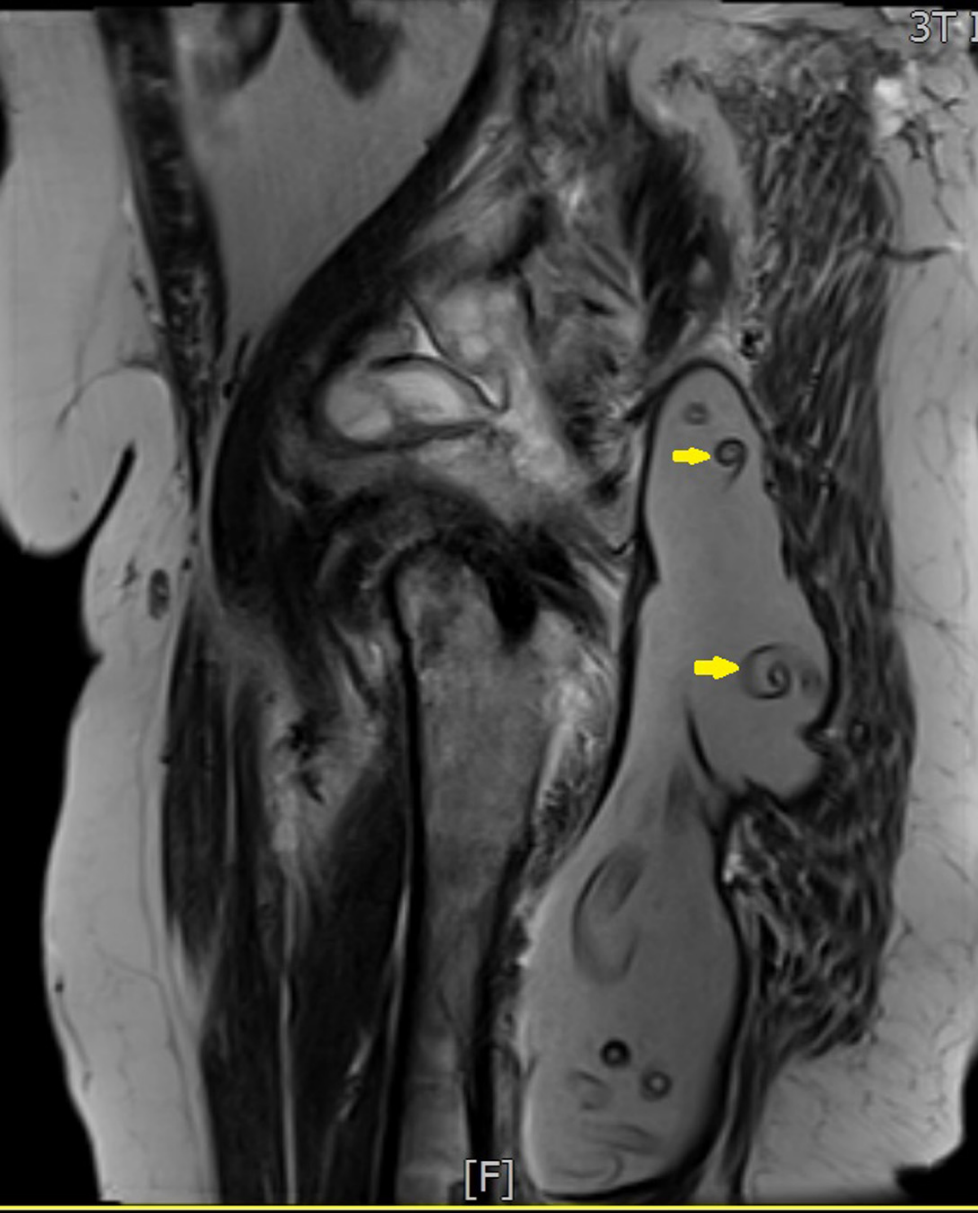
Comma‐shaped lesions (Yellow arrows) within the cyst (sagittal view).

**Figure 4:**
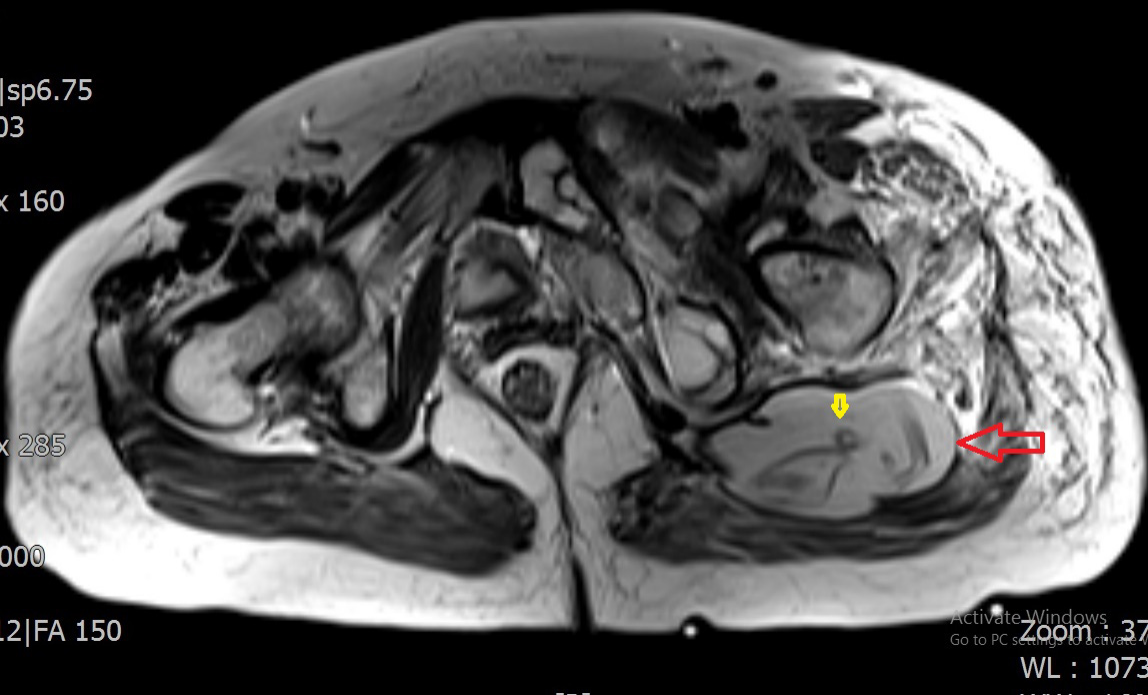
Hydatid cyst (Red arrow) and collapsed membranes (Yellow arrow).

Clinicians should remember to include hydatid cysts in the differential diagnosis of pelvic cysts, especially when radiologic studies identify collapsed cyst walls.

## Conflict of Interest

None declared.

## Authorship

SADM: gave consultation for infectious diseases and involved in manuscript preparation. OR: gathered data and involved in manuscript preparation. SS: gave orthopedic consultation and involved in manuscript preparation. FS: prepared MRI report and gave radiology consultation and involved in manuscript preparation. SMI: prepared pathology slide and involved in manuscript preparation.
